# Behavioral and Electrophysiological Effects of Cortical Microstimulation Parameters

**DOI:** 10.1371/journal.pone.0082170

**Published:** 2013-12-05

**Authors:** Bilal A. Bari, Douglas R. Ollerenshaw, Daniel C. Millard, Qi Wang, Garrett B. Stanley

**Affiliations:** Coulter Department of Biomedical Engineering, Georgia Institute of Technology & Emory University, Atlanta, Georgia, United States of America; Australian National University, Australia

## Abstract

Electrical microstimulation has been widely used to artificially activate neural circuits on fast time scales. Despite the ubiquity of its use, little is known about precisely how it activates neural pathways. Current is typically delivered to neural tissue in a manner that provides a locally balanced injection of positive and negative charge, resulting in negligible net charge delivery to avoid the neurotoxic effects of charge accumulation. Modeling studies have suggested that the most common approach, using a temporally symmetric current pulse waveform as the base unit of stimulation, results in preferential activation of axons, causing diffuse activation of neurons relative to the stimulation site. Altering waveform shape and using an asymmetric current pulse waveform theoretically reverses this bias and preferentially activates cell bodies, providing increased specificity. In separate studies, measurements of downstream cortical activation from sub-cortical microstimulation are consistent with this hypothesis, as are recent measurements of behavioral detection threshold currents from cortical microstimulation. Here, we compared the behavioral and electrophysiological effects of symmetric vs. asymmetric current waveform shape in cortical microstimulation. Using a go/no-go behavioral task, we found that microstimulation waveform shape significantly shifts psychometric performance, where a larger current pulse was necessary when applying an asymmetric waveform to elicit the same behavioral response, across a large range of behaviorally relevant current amplitudes. Using voltage-sensitive dye imaging of cortex in anesthetized animals with simultaneous cortical microstimulation, we found that altering microstimulation waveform shape shifted the cortical activation in a manner that mirrored the behavioral results. Taken together, these results are consistent with the hypothesis that asymmetric stimulation preferentially activates cell bodies, albeit at a higher threshold, as compared to symmetric stimulation. These findings demonstrate the sensitivity of the pathway to varying electrical stimulation parameters and underscore the importance of designing electrical stimuli for optimal activation of neural circuits.

## Introduction

Electrical microstimulation has been used for over a century to better understand the brain’s natural circuitry and to perturb that circuitry to generate percepts [1]. It has been used to probe a wide array of cortical function and connectivity, including networks related to somatosensation [2,3], audition [4,5], vision [6-8], movement [9,10] as well as basic cortical dynamics [11,12]. With regards to the generation of percepts, perhaps the greatest success of electrical stimulation has been the cochlear implant, a device that directly stimulates the cochlear nerve to produce auditory percepts [13]. Electrical stimulation has been used to generate percepts in the somatosensory system [14,15] and extensively in the visual system at the level of the visual cortex [16-18], thalamus [19,20], and more recently in the retina [21,22]. 

Despite its long and varied use, precisely how electrical microstimulation activates neural circuits is not well understood. It has long been recognized that accumulation of charge during microstimulation results in neurotoxicity, resulting in conventional strategies of balanced charge delivery [23,24]. The majority of electrical microstimulation studies have used as the base unit of stimulation a symmetric current pulse waveform, in which the shape of the cathode phase is the same as the shape of the anode phase. Recent *in vivo* work has shown that electrical stimulation does not simply activate cells around the stimulation site as was originally proposed [25] but rather activates axons passing near the electrode tip, resulting in sparse activation of neurons [26,27]. Although electrical stimulation has been successfully used as a surrogate for sensory stimulation in animals [14,20], this mechanism is hypothesized to be the reason that electrical stimulation in humans has occasionally been described as painful, unnatural, or discordant [28-30]. In order to more specifically activate neural tissue and generate more reliable and robust percepts, it is important to develop methodologies to preferentially activate cell bodies over axons. 

Modeling work has shown that altering the time course of the balanced charge delivery (i.e. the current pulse waveform) can indeed shift this balance. While symmetric, cathode-leading, charge-balanced current pulses (referred to as ‘symmetric’) activate a larger proportion of axons relative to cell bodies for most physiologically relevant currents, asymmetric, cathode-leading, charge-balanced current pulses (referred to as ‘asymmetric’) are hypothesized to reverse this bias and selectively activate cell bodies in the central nervous system [31]. Recent *in vivo* work in the rat vibrissa system has shown that this is likely the case. Compared to symmetric current pulses, stimulation of the thalamus with asymmetric pulses led to a cortical response (as measured by voltage-sensitive dye imaging (VSDI)) that was much more similar to the cortical response evoked by whisker stimulation [32]. Specifically, asymmetric current pulses resulted in cortical activation that was more spatially focused than with symmetric current pulses, and more consistent with topographic activation of cortex. A recent behavioral study has further shown that the asymmetric current pulse waveform results in lower current thresholds in cortex for use in brain machine interfaces and neurostimulation devices [33]. What is currently not known, however, is how the symmetry of the current pulse waveform affects behavioral percepts over relevant ranges of current levels and how this directly relates to the cortical activation. 

Here, we delivered single electrical microstimulation current pulses to the barrel cortex of awake, head-fixed, behaving rats trained on a go/no-go task. Animals were trained to detect the presence of single current pulses while both the amplitude and the waveform symmetry were randomly varied from trial to trial. We found that when asymmetric stimuli were applied, a larger current was required to elicit the same behavioral response as compared to symmetric stimuli over a range of current amplitudes, as measured by the probability of a correct detection. In separate experiments in anesthetized animals, we measured the cortical effect of symmetric vs asymmetric cortical microstimulation using VSDI. We again found that for a large range of current amplitudes, a larger current amplitude was required when applying asymmetric stimulation in order to elicit the same cortical response, consistent with behavioral results. Taken together, the behavioral and electrophysiological results here support the hypothesis that delivery of charge in a temporally asymmetric manner may more selectively engage a given neuronal cell body population, resulting in a more controlled cortical activation. 

## Methods

### Ethics statement

Four female Sprague-Dawley rats (Charles River Laboratories, Wilmington, MA; 7 wk of age, ~250 g at beginning of study) were used in the behavioral portion of this study, and five female Sprague-Dawley rat was used for the acute VSDI experiment. Animals were housed on a reversed 12:12-h light-dark cycle, with all experimental sessions occurring during the dark phase. All procedures were in accordance with protocols approved by the Georgia Institute of Technology Animal Care and Use Committee, and were in agreement with guidelines established by the National Institutes of Health.

### Microelectrode Array Fabrication

**Figure 1:**
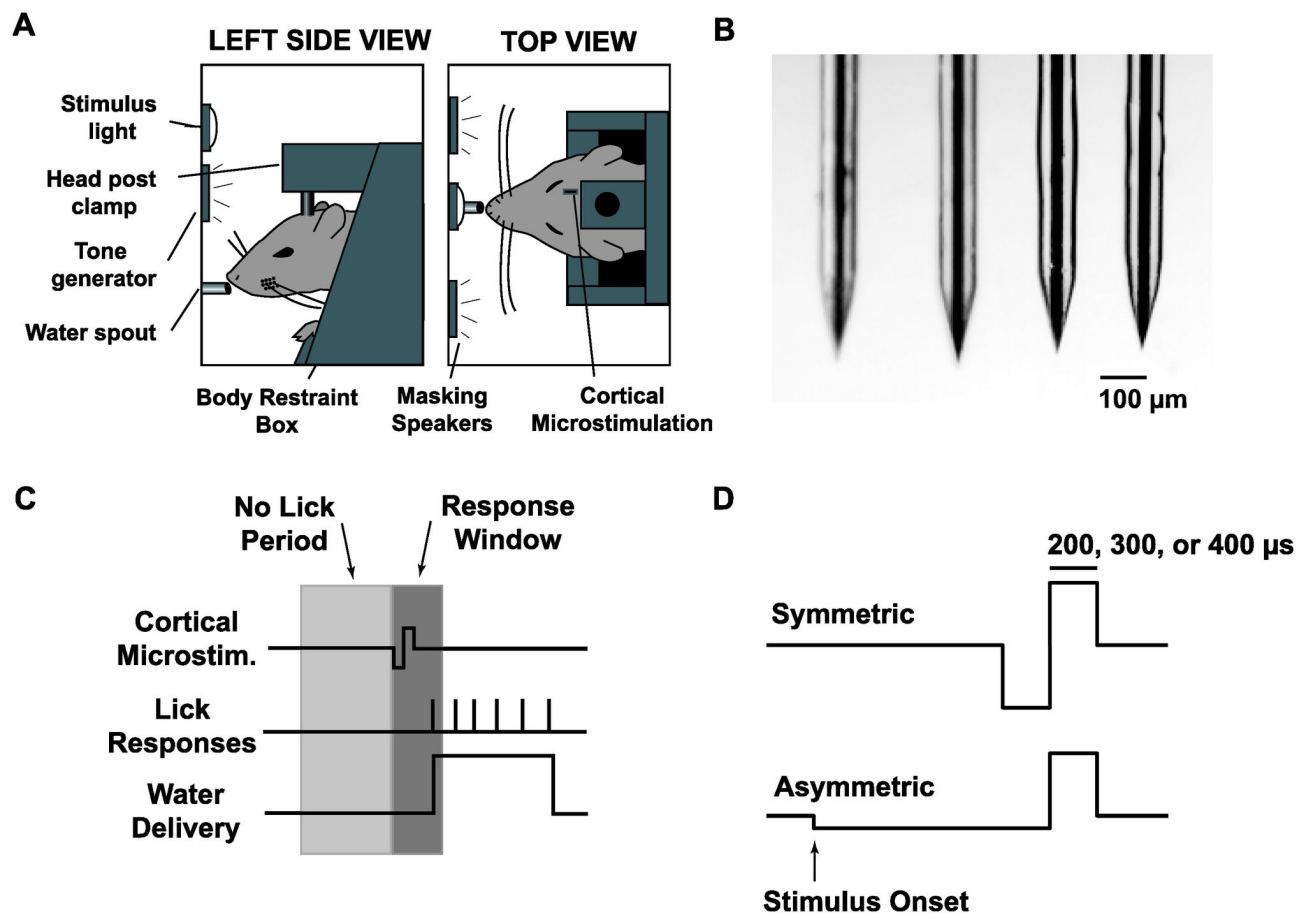
Overview of Go/No-Go Behavioral Detection Task. *A*: Diagram of the behavioral apparatus. Head-fixed animals were placed in a light and sound attenuating chamber and trained to respond to cortical microstimulation by licking a water spout. *B*: Photomicrograph of microelectrode array showing four microelectrodes. *C*: Timeline of the behavioral task. Following the beginning of a trial, cortical microstimulation was presented at a random time drawn from a uniform distribution of 2-8s. To discourage random guessing, a minimum 1s “no lick” period was imposed such that any licks in the 1s preceding stimulus delivery delayed the stimulus onset. Animals had a 0.5s response window following stimulus onset to lick the spout and receive a water reward. Catch stimuli were delivered on 20% of trials, in which no stimulus was delivered, to test for chance response probability. No penalty or reward was given for a response to a catch stimulus. Note that the cortical microstimulation waveform is not drawn to scale. *D*: Microstimulation waveforms. Cortical microstimulation was delivered as either a symmetric current pulse or an asymmetric current pulse. Both symmetric and asymmetric pulses were single pulse, cathode-leading, and charge balanced. Asymmetric pulses had a cathode phase that, relative to the anode phase, was temporally lengthened by a factor of 5 and had amplitude reduced by a factor of 5. For each animal, the duration of the anode phase for both symmetric and asymmetric pulses was fixed at 200, 300, or 400 µs. Stimulus intensity was quantified as the charge delivered per phase of stimulation (in units of nC/phase).

Each animal in the behavioral portion of this study had a 4x1 microelectrode array implanted in the barrel cortex. A 4x1 array was used for redundancy to ensure that a viable microstimulation site was available. Briefly, four polyamide tubes (Miniature Polyimide Tubing, 36 AWG, 0.0050 inch ID, 0.0095 inch OD) were laid side-by-side and embedded in epoxy to create guide tubes for the microelectrodes. These tubes were cut into lengths of 4mm and fixed onto carbon fiber support pieces (6mm length, 3mm width, 0.8mm thickness) using light-curing dental cement (Natural Elegance Flowable composite, Henry Schein). Raw glass-coated tungsten microelectrode wires (Thomas Recording; tungsten diameter of 25µm, glass diameter of 80µm) were cut to lengths of 10mm and ground using a microwire grinder (Thomas Recording). Each microelectrode was soldered to a copper wire (Cooner) and four microelectrodes were threaded through the guide tubes and fixed in place with light-curing dental cement. The copper wires were soldered to a 4x1 connector (Digikey, 0.05 inch pitch male/female header). The microelectrodes on the finished array had an impedance of 100-150 kΩ at 1 kHz and an electrode-to-electrode separation of 200 - 250 µm. A microscope image of the microelectrodes from a finalized array is shown in Figure 1B.

### Head Post and Microelecrode Array Implantation

All surgical procedures adhered to aseptic principles. Methods are described in detail in Ollerenshaw et al. [34]. Briefly, anesthesia was initially induced with isoflurane at 4-5% in the home cage and maintained with a subsequent injection of ketamine/xylazine (50/6 mg/kg), at which point the isoflurane was removed. Anesthesia was maintained throughout the procedure with subsequent injections of ketamine (20% of initial dose). Animals were then placed in a stereotactic device and the scalp was incised. After the skull was cleared of connective tissue, 11 holes were drilled and 1.4mm-diameter stainless steel screws were inserted. These served to anchor the head post, a stainless steel machine screw (M5x20mm) with the threaded end facing upward, to the bone. A craniotomy was performed over the left barrel cortex centered at 2.5mm caudal to bregma and 5.5mm lateral to midline [35] and the microelectrode array was oriented along the rostral-caudal axis. In three of the four animals, barrel cortex was verified with manual deflections of the whiskers using a single electrode to record LFP prior to implantation of the full array. The array was driven to a depth of ~700 µm using a hydraulic micropositioner (David Kopf Instruments, Tujunga, CA) and fixed in place with dental cement. The head post was then held over the midline, and dental cement was applied over the base of the post and skull screws. Following surgery, animals were provided ketoprofen (5 mg/kg) and Baytril (2 mg/kg) and were given a minimum of 5 days of recovery before commencing with behavioral training.

### Water Restriction Schedule

Water restriction was implemented after a minimum of 5 days of recovery after surgical implantation. Training and data recording sessions took place daily, Monday through Friday, and animals did not have access to water in their home cages on those days. Correct responses in the behavioral task were rewarded with 35- to 40-µl aliquots of water and animals were allowed to continue performing the task until sated. The weight of the animal was tracked daily, and, when necessary, water supplements were provided after the daily experimental session in order to maintain the weight of the animal within 90% of its age-adjusted value. Water was provided ad libitum from Friday afternoon through Sunday afternoon of every week and for 1 full week every 2 months.

### Behavioral Apparatus

Animals were placed inside a custom built body restraint box (illustrated in Figure 1A), which itself was placed inside a sound- and light-attenuating cubicle (ENV-014, Med Associates, St. Albans, VT). While on the bench top, animals were guided into the body restraint box and kept in place with a tail plate and a paw restraint plate to prevent excessive movement while the animal was head fixed. A 6-cm aluminum head post extension was attached to the animals’ head post with a set screw and locked onto the body restraint box with two set screws. The body restraint box was subsequently placed in the cubicle. The animal was placed directly in front of a plastic water spout which served to deliver water rewards and to measure licking responses. The water spout was mounted on a lever arm with an embedded piezoelectric sensor. Tongue contact triggered a voltage change which was measured, converted to a binary value, time-stamped, and stored in a data file, thus allowing the onset of each lick of the water spout to be recorded. Water was fed through the spout by a peristaltic pump (Model 80204M, Lafayette Instruments) placed outside the sound attenuating chamber.

### Stimulator Setup

Stimulation waveforms were programmed with MathWorks Simulink and delivered using a dedicated real-time PC running at 50 kHz. The output of the real-time PC was delivered through a linear stimulus isolator (WPI Inc, Sarasota, FL) which allowed a maximum of 100 µA to be sent. This stimulus isolator was directly connected to the animal’s microelectrode connector during the behavioral task.

Cortical microstimulation consisted of a single current pulse that was charge-balanced and cathode-leading. All stimuli were either symmetric or asymmetric waveforms, as shown in Figure 1D. Symmetric stimuli had cathode and anode phases that were equal in amplitude and duration. Asymmetric stimuli had a cathode phase that was temporally lengthened by a factor of 5 and had amplitude reduced by a factor of 5, to maintain charge balance. Due to the maximum 100 µA output imposed by the stimulus isolator, in order to ensure that animals were stimulated with suprathreshold pulses, the stimulus pulse widths were tailored to each animal based on experimentally determined detection thresholds. For example, if it was discovered that a 200 µs, 100 µA pulse was not strong enough to reliably elicit a behavioral response from an animal (>90%), the pulse width was increased to 300 µs. For each animal, the length of the anode phase was manually selected to be 200, 300, or 400 µs and fixed for all subsequent trials. Stimulus intensity was quantified as charge per phase (nC/phase) for analysis.

Control of the behavioral task and data logging were performed with custom software written in Microsoft Visual Basic 6. The animal’s behavioral state was monitored during the task using a low-speed CCD camera (Model DMK 21BF04, The Imaging Source, Charlotte, NC).

### Training and Behavioral Task

After the animal was placed on a water restriction schedule, it was systematically habituated to head fixation and trained to perform the full detection task [34,36,37]. Water was initially hand delivered via a syringe. Animals were slowly habituated to short periods of head fixation until they could remain calm while head-fixed for up to 1 min at a time. The animals were placed in the experimental chamber and for the first two to three sessions, water was delivered continuously, after which a lick of the response spout was required in order to receive a water reward. Once animals could tolerate head fixation for a minimum of 5 min, cortical microstimulation was introduced on one of the four electrodes. Initially, a symmetric pulse (14 nC/phase; 200 µs/phase, 70 µA) was delivered, followed by an automatic delivery of water, to facilitate pairing of cortical microstimulation with water reward. If, after several sessions, the animal did not learn to pair microstimulation with reward, the stimulus intensity was increased by increasing the phase duration to either 300 or 400 µs. Once an association was established, the stimulating electrode channel was fixed and the three other channels were no longer used for the remainder of the study. A minimum 1 s pre-stimulus no-lick window was established and water was only delivered if the animal elicited a lick within a 1.5 s post-stimulus period. Any licks within the pre-stimulus window delayed the stimulus. The post-stimulus period was slowly reduced to 0.5 s and remained fixed for all remaining sessions. Once an animal responded to at least 80% of stimuli, catch trials were introduced to measure chance performance. During catch trials, at the designated stimulus delivery time, no stimulus was delivered. Any licks in the post-stimulus window were recorded and used for analysis but were not penalized. Once an animal responded to at least 80% of stimuli and no more than 20% of catch trials, the full version of the task was employed.

In the full version of the task, shown schematically in Figure 1C, a new trial was initiated with the stimulus delivery time chosen from a uniform distribution of 5 to 8 s. Any lick responses within the pre-stimulus window resulted in a “time-out”, where the stimulus onset was delayed by an additional 1 to 5.5 s. Following stimulus delivery, animals were required to lick during the response window in order to receive a water reward. A trial was categorized as a “hit” if the animal licked the water spout within the response window and a “miss” otherwise. Hits were rewarded with a 35- to 40-µl aliquot of water and misses were not penalized. During the full task, symmetric microstimulation of 8 different intensities and asymmetric microstimulation of 8 different intensities were randomly delivered to generate psychometric data for each animal. Due to individual differences in the threshold of each animal to electrical microstimulation (see Results), a different range of electrical stimuli was chosen for each animal. These values were chosen after 3-5 initial sessions in the full detection task and were selected to ensure that the full range of response probabilities, from chance performance to maximal detectability, was spanned for each animal. The final stimulus parameters for each of the four animals are shown in Table 1.

**Table 1:** Stimulus Parameters for Behavioral and VSDI Animals.

**Animal**	**Anode Phase Duration (µs)**	**Symmetric Stimuli (µA/phase)**	**Symmetric Stimuli (nC/phase)**	**Asymmetric Stimuli (µA/anode phase)**	**Asymmetric Stimuli (nC/phase)**
**Rat 1**	200	[7.5, 15, 22.5, 26.3, 30, 37.5, 45, 55]	[1.5, 3, 4.5, 5.3, 6, 7.5, 9, 11]	[7.5, 20, 27.5, 42.5, 55, 62.5, 75, 90]	[1.5, 4, 5.5, 8.5, 11, 12.5, 15, 18]
**Rat 2, 3**	400	[5, 11.3, 22.5, 28.1, 33.8, 56.3, 62.5, 70]	[2, 4.5, 9, 11.2, 13.5, 22.5, 25, 28]	[11.3, 33.8, 45, 56.3, 67.5, 78.8, 90, 95]	[4.5, 13.5, 18, 22.5, 27, 31.5, 36, 38]
**Rat 4**	300	[5, 10, 17.5, 20, 25, 30, 36.7, 40]	[1.5, 3, 5.3, 6, 7.5, 9, 11, 12]	[7.5, 20, 27.5, 42.5, 55, 62.5, 75, 90]	[2.3, 6, 8.3, 12.8, 16.5, 18.8, 22.5, 27]
**VSDI Data Set 1**	200	[20, 40, 60, 80, 100]	[4, 8, 12, 16, 20]	[20, 40, 60, 80, 100]	[4, 8, 12, 16, 20]
**VSDI Data Set 2, 3, 4, 5**	200	[5, 10, 15, 25, 40]	[1, 2, 3, 5, 8]	[5, 10, 15, 25, 40]	[1, 2, 3, 5, 8]

On every fifth trial, a test stimulus consisting of the second strongest symmetric pulse was presented to probe the attentional/motivational state of the animal. The test stimulus was repeated if the animal failed to respond, and the session was halted if the animal failed to respond to three consecutive test pulses. Catch trials were interleaved on 10-20% of trials. All trials were preceded by a 1-3 s period to ensure separation between individual trials and to ensure that animals had sufficient time to drink the water reward from the previous trial. On a subset of trials, high speed video was used to characterize whisker motion (see Ollerenshaw et al. (2012) for methods).

Animals generally performed one session per day and were allowed to work until sated. In cases in which two sessions were performed in a day, the first session was halted after 15-20 min and the animal waited a minimum of 1 h before starting the second session. Well-trained animals generally performed 100+ correct trials per day. Across all 4 animals, over 5,000 total trials were included in analysis, with each animal being presented each of the 16 possible stimuli (8 intensities of symmetric pulses, 8 intensities of asymmetric pulses) an average of 72 times. 

### Voltage-Sensitive Dye Imaging (VSDI)

Neural data (n = 5) was obtained in a separate set of experiments by measuring the layer 2/3 voltage-sensitive dye response of the cortex of anesthetized animals to putative layer 4 cortical microstimulation with symmetric and asymmetric stimuli. Animals were initially anesthetized with 4% isoflurane before intraperitoneal injection of Nembutal (50 mg/kg) for long term anesthesia. Subsequent doses of Nembutal were used to maintain a surgical level of anesthesia. Animals were mounted in a stereotactic device and a craniotomy was performed over the left parietal cortex (coordinates: 1-4 mm posterior to bregma, 4-7 mm lateral to midline) to expose the barrel representation of the primary somatosensory cortex.

VSDI was used to monitor cortical activation in response to cortical microstimulation. After the craniotomy was performed, the dura was allowed to dry for 15 minutes according to the protocol of Lippert et al. (2007). The cortex was stained with dye RH1691 (1mg/mL; Optical Imaging, Rehovot, Israel) for two hours and subsequently washed for 30 minutes. After washing the cortex, saline was deposited in the cranial window. A 1.0x magnification lens was used in conjunction with a 0.63x condenser lens to provide 1.6x magnification (48 pixels/millimeter). A 150 W halogen lamp filtered at 621-634 nm wavelength was used for imaging the brain surface and for providing excitation of the dye. The VSDI data were acquired at five millisecond interframe intervals (corresponding to a frame rate of 200 fps) beginning 200 milliseconds preceding stimulus presentation. 

A glass coated tungsten microelectrode (impedance = 1-2 megaohms at 1kHz) was advanced into the barrel cortex using a precision microdrive (David Kopf Instruments, Tujunga, CA). The electrode was positioned ~45° from the cortical surface and driven in ~1,000 µm, corresponding to a depth of ~700 µm below the pia, into layer 4 [38]. The stimulus waveforms were designed using a digital stimulus generator (WPI Inc, Sarasota, Florida) and delivered using a current controlled, optically isolated stimulator (WPI Inc, Sarasota, Florida) in conjunction with VSDI. The stimulus parameters are shown in Table 1. 

For each trial, the 40 frames (200 ms) collected before the presentation of the stimulus were averaged to calculate the background fluorescence, against which the activation was measured. For each frame, the background fluorescence was subtracted to produce a differential signal ΔF. Additionally, each frame was divided by the background image to normalize for uneven illumination and staining to produce the signal ΔF/F_0_. For presentation purposes only, the individual trials were averaged together and then filtered with a 9x9 pixel (0.19x0.19 mm) spatial averaging filter. All analyses were performed on the raw (unfiltered) images.

For analysis, the VSDI data were functionally registered to the anatomical map of the barrel cortex in order to discretize the spatiotemporal cortical signal with regard to well-defined cortical columns. The outlines of the barrel cortex columns within a cytochrome oxidase stained tangential slice were created using the Neurolucida software (MBF Bioscience, Williston, VT) and imported into MATLAB (MathWorks, Natick, MA). The functional cortical columns were determined in the VSDI data by deflecting a single whisker using a piezoelectric actuator and recording the cortical response (for methods, see Wang et al. [32]. The initial frame of cortical activation, which has previously been shown to be restricted to a single cortical column [39], was captured for deflection of 4-6 different whiskers during each experiment. Once aligned to the VSD image, the barrel regions were roughly circular and ~20 pixels (0.420 mm) in diameter.

The anatomical mapping from histology was registered with the functional column mapping from VSDI by solving a linear inverse problem, the details of which are described in Wang et al. [32]. Following the functional image registration, the cortical response was discretized, where each signal corresponds to a single functional cortical column. In so doing, the VSDI signal was averaged spatially within the contour of the cortical column. For analysis, only the average ΔF/F_0_ value from the 10 ms poststimulus frame from the stimulated cortical column was presented in this study. However, we also analyzed the data by averaging over time windows of various lengths (20ms, 50ms, 100ms, and 500ms) post-stimulus and found no difference in the results.

### Data Analysis

In analyzing the behavioral data, to prevent the inclusion of trials in which the animal was not highly motivated, trials were excluded from analysis if the animal did not correctly respond to the subsequent test stimulus. Thus a pair of successful responses to test stimuli bracketed each five-trial block. 

For both behavioral and VSDI data, psychometric curves were constructed from the measured responses (either probability of response for behavior, or ΔF/F_0_ for VSD) by fitting a sigmoidal curve of the form P(x) = c + (1 – c)•k/(1+e^-αx-β^) where x is the set of stimulus strengths, c and k set the range (which was set to the min and max of the data), and α and β are free parameters that were calculated with a nonlinear least-squares regression algorithm in MATLAB. For VSDI data sets 2-5, the strongest asymmetric pulse did not elicit a suprathreshold response (strongest asymmetric ΔF/F_0_ was <80% of strongest symmetric ΔF/F_0_). It was assumed that the VSDI response would have saturated at the same level for both symmetric and asymmetric stimuli, had a high enough current been delivered. Under this assumption, the asymmetric sigmoid fits for these data sets were forced to the same saturation level as the symmetric sigmoid fits before analysis. 

**Table 2:** Summary of Calculated Detection Thresholds for the Symmetric and Asymmetric pulses.

**Animal**		**Symmetric Pulse Detection Threshold (nC/phase)**	**Asymmetric Pulse Detection Threshold (nC/phase)**
**Behavioral Animals**	1	5.1	10.7
	2	11.1	23.7
	3	13.9	24.3
	4	6.8	17.5
**VSD Data Sets**	1	7.3	10.8
	2	4.7	10.4
	3	4.1	8.0
	4	2.6	6.0
	5	3.4	7.7

Changes in psychometric curves were quantified as a change in the “midpoint” with the use of asymmetric stimuli over symmetric stimuli. The midpoint was defined as (min(y)+max(y))/2, where y represents probability of response for behavior or ΔF/F_0_ for VSD . Error bars represent 95% confidence intervals for behavioral data and ±1 standard deviation for VSDI data. Confidence intervals on the behavioral data were generated using MATLAB’s “binofit” function, which uses the Clopper-Pearson method to calculate confidence intervals.

## Results

We trained a total of four head-fixed female Sprague-Dawley rats to perform a go/no-go detection task in which single cortical microstimulation pulses were delivered to the barrel cortex [36]. Stimulus waveforms consisted of a single current pulse that was charge-balanced, cathode-leading, and either symmetric or asymmetric in shape. Symmetric stimuli had cathode and anode phases that were equal temporally and in amplitude. Asymmetric stimuli had a cathode phase that, relative to the anode phase, was temporally lengthened by a factor of 5 and had amplitude reduced by a factor of 5 (see Methods and Figure 1D). Consistent with previous results, animals rarely moved their whiskers in the experimental conditions used here [36,40,41]. The paucity of active whisker movement means that stimuli were likely delivered while the barrel cortex was in the ‘passive state,’ characterized by high sensory responses and low background firing [42]. The behavioral task is described in detail in Methods and is shown schematically in Figure 1. 

### Detection Performance in the Go/No-Go Task

**Figure 2:**
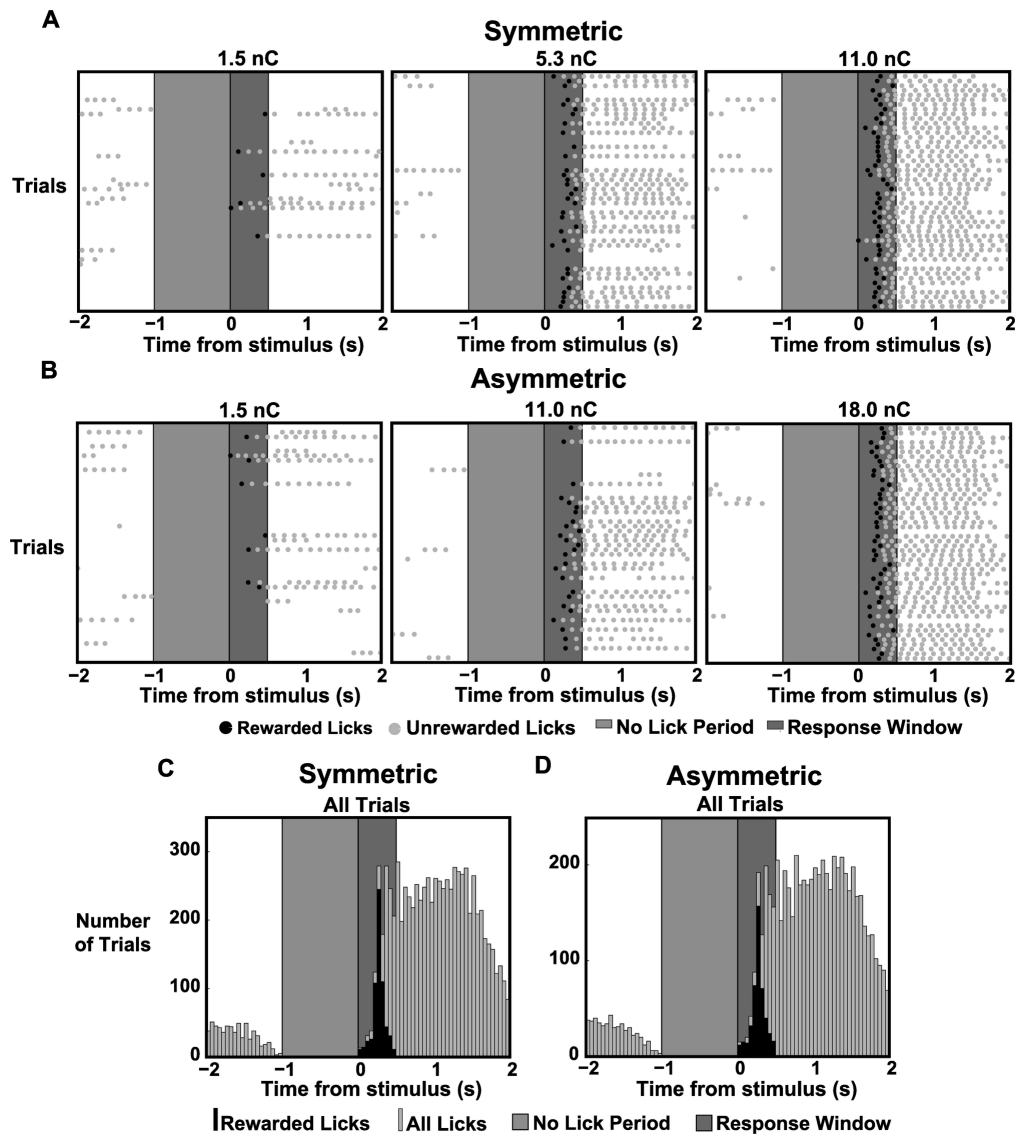
Lick Response Raster Plots and Histograms for Symmetric and Asymmetric Stimulation. *A*,*B*: Lick response raster plots for a single animal (rat 1) with 50 trials shown for three symmetric stimulus intensities and three asymmetric stimulus intensities. The light gray region indicates the minimum 1s “no-lick” period while the dark gray region indicates the 0.5s response window. Tick marks indicate tongue contact with the water spout with black tick marks indicating a response for which a reward was given. *C*,*D*: Histograms from the same animal showing lick responses to all trials for both symmetric and asymmetric stimuli. The black histogram indicates rewarded licks while the gray histogram indicates all licks.

The four animals in the behavioral portion of the study performed a total of over 5,000 trials which were included in the analysis. Each animal received an average of 72 presentations of each of the 16 possible stimuli (8 symmetric stimuli, 8 asymmetric stimuli). Figure 2A,B shows lick response rasters for 50 trials for three symmetric stimuli and three asymmetric stimuli for rat 1. The light gray portion in the lick rasters designates the minimum length of the enforced no-lick period, during which any licks emitted by the animal resulted in an additional randomized delay of the stimulus. This no-lick period was designed to prevent the animals from licking impulsively. The dark gray section of lick rasters represents the 0.5 s response window, during which the animal was required to respond to receive a water reward. Each tick mark in the lick raster represents the time of contact of the animal’s tongue with the water spout. The first lick after the stimulus within the 0.5 s response window resulted in a water reward for the animal and is highlighted (rewarded lick, black). It should be noted that stimuli were delivered at random times for each trial and are artificially aligned for visualization. Subsequent licks were generally a result of the animal consuming the water reward (unrewarded lick, gray). The three symmetric pulses (1.5, 5.3, and 11.0 nC/phase) show an increase in correct responses with increasing stimulus strength. The same trend holds true for the three asymmetric pulses (1.5, 11.0, 18.0 nC/phase). Qualitatively, it can also be seen that the 11.0 nC/phase symmetric pulse shows more correct responses than the 11.0 nC/phase asymmetric pulse. In general, it takes a stronger asymmetric pulse to obtain the same response probability as a symmetric pulse. Figure 2C,D shows the lick response for rat 1 for all symmetric and asymmetric trials, respectively. It can be seen that the timing of the animal’s response does not differ between the two types of stimuli. 

During training, the threshold for detection was ascertained and found to differ from animal to animal based on slight differences in electrode implantation depth and location. In order to employ a range of stimuli wide enough to include both chance performance and suprathreshold performance, but narrow enough to capture details in between the two extremes, each animal had stimulus parameters individually set. For example, the symmetric stimulus set for rat 1 ranged from 1.5 to 11.0 nC/phase, while the symmetric stimulus set for rat 2 ranged from 2.0 to 28.0 nC/phase.

**Figure 3:**
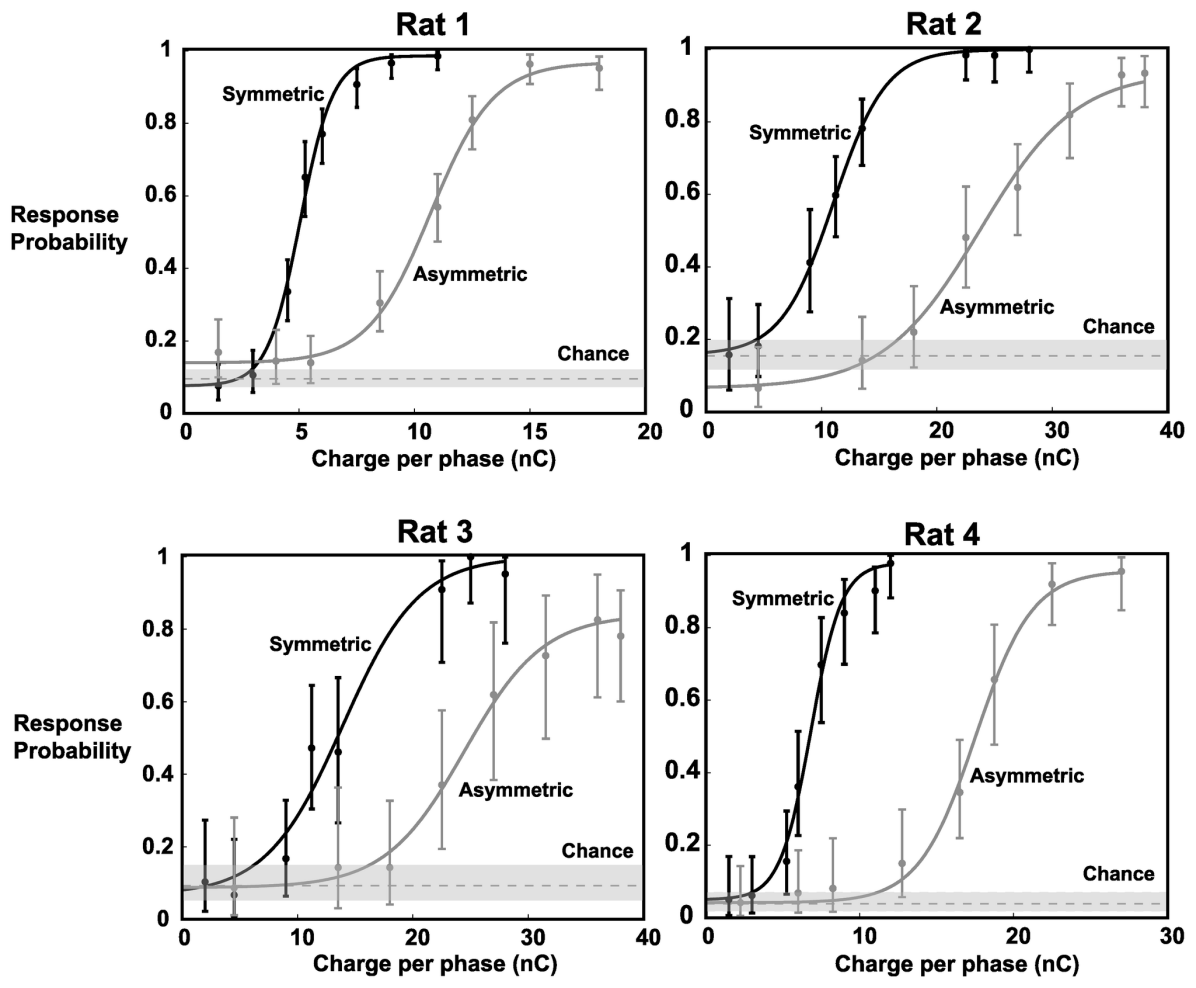
Behavioral Results of Go/No-Go Detection Task. Psychometric curves for each of the 4 animals indicate an increase in detection threshold with the use of asymmetric pulses. Solid lines represent sigmoidal fits to the response probabilities for the 8 tested symmetric pulse intensities and the 8 tested asymmetric pulse intensities. Chance was measured as the response probability to catch stimuli. With the use of an asymmetric pulse, there was a rightward shift to the psychometric curve, quantified by an average increase of 113.9% in the midpoint (defined as the average between the min and max values). Note different scales for the x-axes for different animals. Error bars represent 95% confidence intervals.

Figure 3 shows the individual response probabilities as a function of stimulus intensity (in units of nC/phase). Each of the four animals showed low response probabilities (between 4 and 17%) for the weakest stimuli and plateaued at a maximum for the strongest stimuli, typically >90%. Previous results, in our lab and others, have shown that this is typical performance in similar go/no-go tasks [34,36,41]. The dashed gray line represents the response probability to catch stimuli, an estimate of chance performance, which varied between 4% and 15%. For all animals, the responses to the lowest amplitude stimuli fall within chance performance, implying that animals were employing a guessing strategy. With the use of asymmetric stimuli, sigmoidal fits for all animals show a rightward shift. This means that, for the same stimulus strength, asymmetric pulses are less detectable and need to be increased in intensity to obtain similar response probabilities.

### Voltage-Sensitive Dye Imaging (VSDI) of Cortical Activation

**Figure 4:**
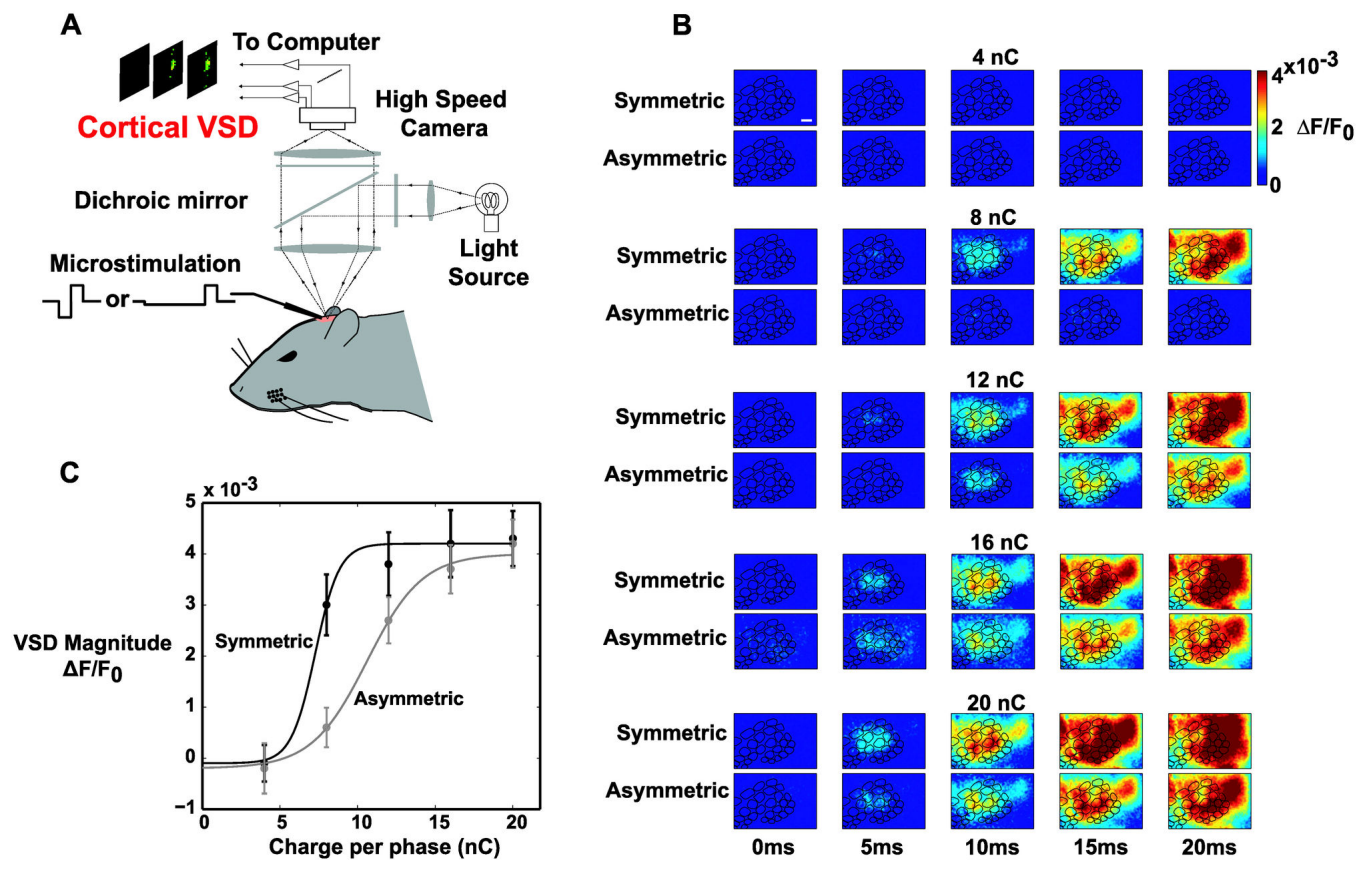
Voltage-sensitive Dye (VSD) Imaging in Response to Cortical Microstimulation. *A*: Schematic of the VSD setup. A craniotomy was performed over the barrel cortex and VSD RH1691 was allowed to diffuse into the cortex. A microelectrode was then driven ~700 um below the pia and a high-speed camera was focused 300 µm below the pia. Following cortical microstimulation, images of the cortical surface were captured every 5ms (200 frames per second). *B*: Representative image frames (VSDI Data Set 1) showing the spatiotemporal evolution of the VSD signal in response to symmetric and asymmetric microstimulation of various intensities. The ΔF/F_0_ value in the 10ms frame was averaged in the stimulated barrel for analysis. Scale bar is 500 µm. *C*: Representative neurometric data (VSDI Data Set 1) revealed a rightward shift in the stimulus-response curve with the use of an asymmetric pulse. Solid lines represent sigmoidal fits to the response probabilities for the five tested symmetric pulse intensities and the five tested asymmetric pulse intensities. The use of asymmetric pulses resulted in a rightward shift of the neurometric curve, quantified by a 47.9% increase in the midpoint. These findings parallel the behavioral results. Error bars represent ±1 standard deviation.

In separate experiments in anesthetized animals, VSDI was used to characterize the population cortical response resulting from cortical microstimulation with the symmetric and asymmetric pulses used during the behavioral task. VSDI has previously been shown to capture primarily subthreshold membrane potential fluctuations in layer 2/3 pyramidal neurons [39,43,44]. Figure 4A shows a schematic of the VSDI setup. A microelectrode was placed ~700 µm below the pia and 5 different currents were delivered 10 times each for symmetric and asymmetric pulses (see Table 1). Images of the resulting layer 2/3 cortical activation were captured at 200 Hz, with each stimulus applied 10 times total. For purposes of analysis, a barrel map was registered to the cortical image (see Methods) and the magnitude of the VSD signal was measured in the stimulated barrel. 

Figure 4B shows one representative example of the spatiotemporal evolution of the VSD signal in response to symmetric and asymmetric pulses of various intensities for which some qualitative observations can be made. For this animal, with very low stimulus intensities (4 nC/phase), no VSD signal could be discerned. At 8 nC/phase, the symmetric pulse caused large widespread activation while the asymmetric pulse resulted in a more constrained and lower amplitude response. As the pulse strength was increased, symmetric pulses consistently resulted in greater overall activation, both in amplitude and spatial extent. At 20 nC/phase, the VSD response begins to look more similar, although symmetric pulses consistently activated more cortical surface area in the 10ms window. This response profile was typical of all the VSD data sets.

The ΔF/F_0_ value was averaged in the stimulated barrel at the 10ms post-stimulus timepoint to generate the neurometric data for this animal, shown in Figure 4C. This representative data shows that, with the use of asymmetric pulses, there is a rightward shift in the sigmoid fit for the VSDI data. This trend holds for all the VSD data sets. This parallels the behavioral findings (Figure 3) in which a higher behavioral threshold was found using asymmetric stimuli.

Table 2 summarizes the change in the threshold based on the sigmoid fits for both symmetric and asymmetric stimulation for both psychometric and neurometric data sets, in which the threshold was calculated as the average between the minimum and maximum values of the sigmoidal fit. There was an average increase of 113.9% in the midpoint of the behavioral dataset (n=4) and an increase of 104.3% in the neural dataset (n=5). This shows that asymmetric pulses resulted in a rightward shift in both behavioral and neural sigmoid fits. This rightward shift means that a stronger asymmetric pulse is needed to obtain the same level of detectability relative to a symmetric pulse. 

## Discussion

Activation of specific volumes of neural tissue is critical both for uncovering functional connections and for generation of specific percepts for neural prosthetic applications. The majority of stimulation protocols involve high frequency trains of current pulses. The most commonly used pulse waveform, the symmetric, cathode-leading pulse, has been shown to preferentially activate axons near the electrode tip, resulting in sparse activation of neurons [27]. Altering the waveform shape may reverse this bias and result in preferential activation of local cell bodies, a hypothesis that was originally based on modeling data [31] and recently supported *in vivo* [32]. The original modeling work predicted that asymmetric stimuli, identical to those used in this study, would preferentially activate local cell bodies over axons through manipulation of nonlinear properties of sodium channels. The initial long duration cathodal pulse is believed to cause the opening of sodium channels at the axons, but not to the degree that an action potential is generated. Thus, when the short-duration anodal pulse follows, the sodium channels in the axons are inactivated and unable to re-open, leaving only the cell bodies to respond. By fundamentally altering the composition of neural elements activated by electrical stimulation, asymmetric stimuli stand to generate distinct downstream responses, and ultimately shape perception in a more controllable manner.

Here, we characterized both the psychometric and neurometric effects of electrical microstimulation delivered to barrel cortex. Specifically, we designed a behavioral go/no-go task in which head-fixed rats were stimulated with a single cathode-leading current pulse that was either symmetric or asymmetric in shape. We designed the experiments using a single pulse instead of a train of pulses to remove potential effects related to frequency and timing of stimulation. We found that when asymmetric stimuli were applied, a larger current was required to elicit responses on the same proportion of trials as compared to symmetric stimuli, as reflected in the rightward shift of the psychometric function in response to asymmetric stimuli. In contrast to a pure scaling along the behavioral response axis where the performance for the symmetric and asymmetric stimuli would plateau at different levels, however, we found that the peak performance was identical for the different stimulus classes, suggesting a shifting of performance along the current axis. In separate experiments in anesthetized animals, we used voltage-sensitive dye imaging to characterize the neural response to cortical microstimulation and found a similar trend. Thus, the reduced behavioral detectability resulting from asymmetric stimuli appears related to the higher current required to activate a given neural population. It should be noted that our analysis does not differentiate between whether this increase was due to a rightward shift in the curves or due to gain modulation. Psychophysically, the change in the threshold stimulus intensity is most directly relevant. Biologically, however, the exact relationship between the neuronal response and stimulus intensity may provide more information on the mechanism of asymmetric electrical stimuli. A gain modulation, or direct scaling of the input strength, would be consistent with the hypothesis that the long duration cathodal phase reduces the excitability of nearby neuronal elements by inactivating sodium channels. Regardless of the mechanism, the observed increase in threshold with the use of asymmetric stimuli remains.

The early onset of layer 2/3 activity measured through voltage sensitive dye imaging is thought to primarily represent activity in the corresponding layer 4 cortical column [39], where our stimulating electrode was positioned during the VSDI experiments. Primary cortical activity has been shown to be necessary for whisker based tasks [45], thus indicating that activity in S1 represents a critical step in ultimately forming a percept. The reduced magnitude of layer 2/3 activation in response to asymmetric stimuli indicates that the increased behavioral thresholds are likely a direct result of increased neuronal thresholds. 

It is important to note that we did not see a more localized response in the VSD response to asymmetric stimuli compared to symmetric stimuli. Intuitively, it would seem that stimulating cell bodies, and not fibers of passage, would lead to a more localized cortical response. Indeed, previous anesthetized work in our laboratory has shown that this is the case for thalamic microstimulation when the downstream cortical activity is measured with voltage-sensitive dye imaging [32]. We believe the distinction between our previous results and those presented here is likely explained by the differential anatomy in the thalamus and cortex. In our previous work, based on the orientation of the thalamus, our electrode was likely activating axons from other “barreloids” creating a greater spread in the cortical response by recruiting adjacent cortical columns. However, here, in layer 4 of cortex, where our electrodes were positioned, the axonal connections almost exclusively project to other cortical layers within the same column. In this case, the stimulation of axons might lead to faster (or irregular) propagation across cortical layers, but might not be expected to cause a greater spread of activation. The stimulation of axons projecting up and down the principal cortical column may additionally help to explain the differences in detectability between the symmetric and asymmetric stimuli. It may also be the case that the VSDI technique does not have the appropriate resolution in this case to measure changes in spatial spread, particularly if the spread is characterized by a sparse activation of individual cell bodies as seen previously using two-photon imaging [27]. Future work should further investigate the effects of asymmetric stimulation on spatial spread within cortex and the nonlinear dynamics recruited by patterned electrical stimulation.

The current study was inspired by several other recent related behavioral studies. A related study by Koivuniemi and Otto [33] tested the effect of a wide range of parameters, including waveform symmetry, on stimulus detectability in response to microstimulation in the auditory cortex. In contrast to this study where the behavior was tested in the context of two waveform patterns over a range of current amplitudes, Koivuniemi and Otto’s study reported threshold effects of very wide range of stimulus pulse designs. Most pertinent to the current study, their data suggest that the duration of the cathodal phase of the current pulse was a key determinant in detectability. It is important to note, however, that in addition to the range of stimulus parameters studied, there are a number of significant differences in the studies. Due to the wide range of parameters tested in their study and the limited number of trials possible with their behavioral paradigm, they focused on measuring threshold alone, as opposed to characterizing the psychometric curve across the full range of currents. However, our primary result that a cathode-leading asymmetric pulse leads to an increase in detection threshold is entirely consistent with their findings, though our absolute detection thresholds (5-14 nC/phase and 10-25 nC/phase for symmetric and asymmetric stimuli, respectively) are somewhat lower than theirs (>10 nC/phase for symmetric stimuli and >70 nC/phase for asymmetric stimuli). In another study by Butovas and Schwarz [36], rats were stimulated in the somatosensory cortex to assess the effects of the stimulus intensity, number of pulses, and frequency on detectability. Although they were able to obtain very high response probabilities with two or more pulses, their maximum response probability for single pulses plateaued at ~80%. Here, responses to strong single pulses regularly exceeded 90%. Additionally, their detection thresholds were much lower (2.07 ± 0.4 nC for single pulses) than those measured here, though this difference might be explained by differences in implantation depth, given that their electrodes were implanted to approximately 1500 μm. It should also be noted that the range of stimuli delivered varied from animal to animal and was individually tailored during training. For example, the symmetric stimulus set for rat 1 ranged from 1.5 nC/phase to 11 nC/phase, while it ranged from 2 nC/phase to 28 nC/phase for rat 2. It has been shown that there is generally a decrease in stimulus threshold with increasing depth in the cortex of rats [46]. Although care was taken to insert each electrode to a depth of ~700 µm, it is possible that slight shifts may have occurred during recovery. Additionally, there were slight variations in the lengths of the four electrodes used to make each electrode array, potentially resulting in slight depth variations. It should be noted that previous studies have also noted variations in thresholds across animals of a similar order of magnitude [33,47,48]. We also noted a small but nonsignificant decrease in response time with increasing stimulus strength with both symmetric and asymmetric stimuli (data not shown). This is in contrast to behavioral studies in which the whisker is directly stimulated, where a significant difference in response time was seen between weak and strong stimuli [34,49]. This difference could be explained by the direct stimulation of cortex in our task, which bypasses several afferent sensory processing stages, which would result in a smaller change in reaction time.

In the context of the behavioral experiments presented here, it cannot be determined whether the different stimulation waveforms resulted in percepts that just differed in intensity or generated qualitatively different sensations. However, aside from the reduction in response probability for a given stimulus amplitude, there appeared to be no systematic difference in the way that rats treated symmetric and asymmetric pulses. Latency to lick was similar for both symmetric and asymmetric stimuli (data not shown) and, in general, animals responded to >90% of the strongest symmetric and asymmetric pulses. Additionally, great care was taken to reduce as many confounding variables as possible. We chose to deliver single pulses instead of trains of pulses to remove any confounds that may result from frequency of stimulation. Pulse durations, once set for a given animal, were fixed for all subsequent experiments, to remove the effects of changing the duration of the stimulus. Stimulus strengths were chosen to cover the entire behaviorally relevant detection axis, from chance response, to threshold response, to suprathreshold response. Given the simplicity of the detection task utilized here, any differences in percepts may not directly affect the behavioral outcome, and more complex discrimination tasks would be required to uncover any differences in sensations beyond that related to magnitude. Electrical stimulation in humans has been shown to lead to unnatural or discordant stimuli [28-30], where the large majority of techniques involve symmetric current pulse delivery. The reported discordant/diffuse effects could thus be potentially attributed to the non-selective activation of axons of passage, but further investigation is required to fully understand the perceptual consequences of the phenomena described here.

In the context of neuroprostheses, stimulation with higher currents can be a concern since it has been shown that prolonged stimulation can damage neural tissue [23]. Charge-balanced pulses have been shown to have a damage threshold at up to 300 nC/phase [24]. Since our strongest current intensities were an order of magnitude lower, it is likely that no damage was caused by our stimulus intensities in this study. In a neuroprosthetic application, given that stimulus intensities will likely be much lower than 300 nC/phase, electrical damage to neural tissue is improbable. While the asymmetric stimuli will lead to greater overall power consumption, which could be concern for implantable devices, this could be an acceptable tradeoff for the potentially improved spatial specificity.

Electrical microstimulation has long been known to suffer from lack of specificity, as described here, as well as non-selective activation of different cell types. The recent advent of optogenetic techniques for genetically targeting specific cell types may alleviate some of these issues, but may still suffer from indiscriminate activation of cell bodies and axons, and electrical microstimulation currently remains the only clinically viable means by which to activate neurons on fast time scales. In any case, more precise activation of neurons is critical to uncover the details of neuronal circuit function and to generate robust and discriminable percepts. Although the behavioral work here focused on simple detection tasks, the electrophysiological results here and in our previous work [32] suggest that careful design of the base unit of microstimulation is requisite for generating sets of discriminable stimulus patterns for prosthetic applications. The space of discriminable stimulation patterns across electrode arrays would strongly depend upon the ability to deliver robust spatially localized activation, and thus the parameters of the charge delivery serve as a key element in the optimal design of surrogate inputs in neural pathways.
